# Older Adults’ Outdoor Walking and Inequalities in Neighbourhood Green Spaces Characteristics

**DOI:** 10.3390/ijerph16224379

**Published:** 2019-11-09

**Authors:** Razieh Zandieh, Javier Martinez, Johannes Flacke

**Affiliations:** 1 Department of Planning and Environmental Management, University of Manchester, Oxford Road, Manchester M13 9PL, UK; 2 Faculty of Geo-Information Science and Earth Observation (ITC), University of Twente, P.O. Box 217, 7500 AE Enschede, The Netherlands; j.martinez@utwente.nl (J.M.); j.flacke@utwente.nl (J.F.)

**Keywords:** open space, park, GIS, GPS, physical activity, walkability, socioeconomic, hierarchical analysis, healthy urban planning, urban design

## Abstract

Outdoor walking has considerable benefits for healthy ageing and older adults are recommended to walk regularly. However, older adults living in high-deprivation areas walk less than those living in low-deprivation areas. Previous research has shown that the characteristics of neighbourhood green spaces (i.e., proximity, attractiveness, size, and number) may influence outdoor walking. This study examines spatial inequalities in the characteristics of neighbourhood green spaces in high- versus low-deprivation areas and their possible influences on disparities in older adults’ outdoor walking levels. For this purpose, it included a sample of 173 participants (≥65 years) and used secondary data and a geographic information system (GIS) to objectively measure neighbourhood green spaces characteristics. Geographic positioning system (GPS) technology was used to objectively measure outdoor walking levels. Data on participants’ personal characteristics were collected by questionnaire. The results indicate that one characteristic of neighbourhood green spaces (i.e., size) is positively related to outdoor walking levels. They show that inequalities in neighbourhood green spaces’ size in high- versus low-deprivation areas may influence disparities in older adults’ outdoor walking levels. Despite inequalities in other neighbourhood green space characteristics (e.g., proximity, attractiveness, and number) in high- versus low-deprivation areas, no relationship was found between these neighbourhood green space characteristics and participants’ outdoor walking levels. Enhancing the distribution or creation of large neighbourhood green spaces (e.g., through creating green space networks) may enhance outdoor walking among older residents, especially in high-deprivation areas.

## 1. Introduction

Outdoor walking is a form of physical activity that includes walking for transport, recreation, and exercise. It has substantial benefits for healthy ageing [1,2]. It reduces risks of chronic disease—such as heart disease, type 2 diabetes, and elevated cholesterol—that are prevalent in later life [3,4]. It also improves social interactions and reduces the risk of loneliness [2,3]. While all older adults are recommended to walk [5,6,7], some older adults adopt a less active lifestyle. It has been shown that older adults living in high-deprivation areas (urban areas with high levels of socioeconomic disadvantage) spend less time walking outside their homes than those living in low-deprivation areas [8,9].

The reason behind disparities between outdoor walking levels (duration of walking outside home) of older adults living in low- and high-deprivation areas is mainly unexplored. Nevertheless, evidence shows that neighbourhood built environment elements (e.g., safety, aesthetics, and certain types of land use such as green space) have a critical role in encouraging older adults to do physical activity, such as walking [4,10,11]. Accordingly, one potential explanation for disparities in older adults’ outdoor walking levels is inequalities in neighbourhood built environment elements in high- versus low-deprivation areas. Urban planners should consider this hypothesis in the context of addressing ‘spatial inequality’; that is, the uneven provision of urban resources among urban areas with different levels of socioeconomic disadvantages [12]. The influence of spatial inequalities in some neighbourhood built environment elements (e.g., safety, quietness, land use intensity) on disparities in older adults’ outdoor walking levels has been examined in a few past studies [13,14,15,16]. However, neighbourhood green space characteristics have been rarely addressed in these studies.

Neighbourhood green space is a key built environment element for encouraging outdoor walking among people [17,18,19]. It refers to a local open space with a high degree of cover by vegetation that is accessible to all people at no cost, such as a public park [20]. It is a setting in which people take recreational walks, it is an interesting destination that persuades people to walk to them, and it is a part of a route that people take to reach another destination [17,21]. Walking in neighbourhood green spaces increases contact with nature and has restorative effects and psychological benefits, particularly for older adults [2]. Enhancing neighbourhood green spaces is potentially an effective way to encourage walking among people [22]. Previous research has shown that intensity of neighbourhood green spaces (the amount of land devoted to all green spaces in a neighbourhood) is positively related to higher levels of outdoor walking among older adults [14,23].

In addition to intensity, four characteristics of neighbourhood green spaces—proximity, attractiveness, size, and number (defined in Table 1)—may influence outdoor walking levels. These neighbourhood green spaces’ characteristics are important for the users and potential users of green spaces [24]. It is known that use of neighbourhood green space is sensitive to proximity [18,25,26]. Close proximity to a neighbourhood green space encourages walking for transport by offering a short walking distance to the green space, as a destination. A review of 50 quantitative studies found that proximity to green space is generally related to greater physical activity, such as walking [18]. However, qualitative evidence indicates that close proximity alone may not be enough to encourage older adults to walk [14,27]; therefore, considering the attractiveness, size, and number of neighbourhood green spaces is important. Attractive neighbourhood green spaces make walking pleasant by providing a variety of scenery; a connection with nature, light, and facilities (e.g., café); and safety [14,24], as well as increasing time spent for recreational walking [28]. Large green spaces tend to have more features that provide more satisfying experiences for users [24] and a larger number of gates [14] that facilitate getting to a neighbourhood green space via different routes. A larger number of green spaces in a neighbourhood provides a greater number of destinations and opportunities for outdoor walking. Previous research on the relationships between green space characteristics and physical activity has reported mixed results [29]. These variations are probably the result of different conceptual and methodological approaches (e.g., definition of green space; (context free) measure of physical activity; different methods for assessing green space characteristics, such as attractiveness) applied in previous studies [17]. Nevertheless, some previous studies have shown that the attractiveness and size of green spaces [24,30], as well as the number of neighbourhood green spaces [19,26], are positively associated with levels of physical activity or walking.

An expanding body of literature has explored relationships between neighbourhood green space characteristics and physical activity, including walking [22,24,28,33,34,35], with the following limitations: (1) only a few studies have focused on older adults’ outdoor walking levels [22,28]; (2) previous research has mainly used a self-reported measure of physical activity or walking, which may not be the same as people’s actual walking levels [22,24,28,33,34,35]; and (3) most previous studies on physical activity and walking have addressed single neighbourhood green space characteristics, such as proximity [36,37,38,39,40]; therefore, the effects of other neighbourhood green space characteristics (e.g., size and attractiveness) of the (e.g., nearby) green space have been ignored [17,41]. Focusing on a particular characteristic of neighbourhood green space (e.g., size) may be inadequate for evaluating the impact of neighbourhood green spaces characteristics on outdoor walking, because it cannot distinguish if the size of large green spaces, or attractiveness or proximity of large green spaces, encourages outdoor walking. Therefore, Sugiyama et al. [30] have adopted a specific approach in their research on adults’ walking: they have studied characteristics (i.e., proximity, attractiveness, and size) of three types of neighbourhood green spaces (i.e., the closest, most attractive, and largest) and have found that having larger attractive green spaces (but not necessarily close) may help adults to achieve a sufficient amount of recreational walking. To date, this approach has rarely been applied in studies on older adults’ outdoor walking; and (4) the context of spatial inequality has been rarely addressed in these studies. While evidence shows inequalities in neighbourhood green space characteristics between areas with different levels of deprivation [31,42,43], only a few studies of adults’ physical activity have addressed inequalities in neighbourhood green space characteristics [44,45], or have considered area deprivation as a covariate in analysis [24,46]. Therefore, given disparities in older adults’ outdoor walking levels, a critical question has remained unanswered: To what extent do inequalities in neighbourhood green space characteristics in high- versus low-deprivation areas drive disparities in older adults’ outdoor walking levels?

The question outlined above is critical for urban planning and design, as these disciplines aim to create healthy and equitable cities that encourage and support all citizens’ outdoor walking [47]. Answering this question assists urban planners and designers to identify inequalities in the distribution and design of neighbourhood green spaces that support older adults’ outdoor walking and highlights shortcomings in high-deprivation areas.

Therefore, this study addresses the limitations of previous studies and aims to examine inequalities in neighbourhood green spaces characteristics in high- versus low-deprivation areas and their possible influences on disparities in older adults’ outdoor walking levels. It uses objectively measured outdoor walking levels and green space characteristics and answers two research questions: (1) How (un)equal are neighbourhood green spaces characteristics (i.e., proximity, attractiveness, size, and number) in high- versus low-deprivation areas? (2) What are the relationships between neighbourhood green spaces characteristics (i.e., proximity, attractiveness, size, and number) and older adults’ outdoor walking levels in low- and high-deprivation areas? To answer these research questions, this study applies the approach used in Sugiyama et al.’s study [30] in this way: it identifies three types of neighbourhood green spaces that people may usually use: the closest, most attractive, and largest neighbourhood green spaces [30]. Then, it studies characteristics (i.e., proximity, attractiveness, and size) of these three types of neighbourhood green spaces in low- and high-deprivation areas. Investigating multiple neighbourhood green spaces helps to more accurately examine the influence of neighbourhood green spaces characteristics on older adults’ outdoor walking in low- and high-deprivation areas [30]. The number of neighbourhood green spaces will be studied irrespective of the three types of green spaces. The study is controlled for older adults’ individual characteristics, as evidence shows relationships between older adults’ individual characteristics (e.g., marital status and ethnicity) and their walking levels [13,48,49].

## 2. Materials and Methods

**Figure 1:**
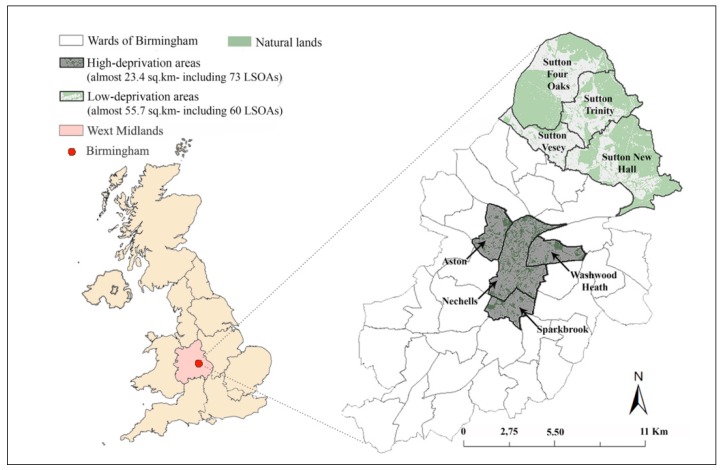
Left: location of Birmingham in the United Kingdom (the United Kingdom map adapted from the work of [53]); right: locations of low- and high-deprivation areas in Birmingham. Adapted from the work of [13].

This study was conducted in Birmingham, United Kingdom (U.K.), shown in Figure 1. Birmingham is a superdiverse city [50] with a population of over one million inhabitants. This study used the sample (*n* = 173) data on outdoor walking levels (measured using geographic positioning system (GPS) technology), and data on individual characteristics (collected by completing a questionnaire) from a previous study [13]—these data were collected from 7th July to 31st October 2012. We measured neighbourhood green spaces characteristics using secondary data (Table 2) and a geographic information system (GIS). Detailed information on measuring neighbourhood green spaces characteristics is presented later in this study.

### 2.1. Identifying Low- and High-Deprivation Areas

To identify low- and high-deprivation areas on an electoral ward scale [51], the index of multiple deprivation (IMD) was used [13]. This index is a U.K. measure of seven main types of deprivation (i.e., income, employment, health and disability, education and skills, barriers to housing and services, crime, and living environment) and is produced at level of lower layer super output areas (LSOAs), which are homogenous small areas of relatively even size with approximately 1500 residents [52]. The deprivation level of each ward was calculated based on the area of each ward that is covered by the 20% most or 20% least deprived LSOAs [13]. Thus, four low-deprivation areas (located in northern part of Birmingham) and four high-deprivation areas (located in inner part of Birmingham) were identified (Figure 1).

### 2.2. Participant Recruitment

For participant recruitment, convenience sampling was used, as it is often the norm in studies on older adults’ health behaviour [54]. Participants were recruited from social centres, such as community centres and University of the Third Age, from the eight selected wards [13]. Maximum similarity to ethnic diversity of each ward’s population was obtained using quota sampling and U.K. census data (2001). Participants were informed about the research and process of participation in the research through advertisements and information sessions in social centres. They signed a consent form before participating in the study. All participants (*n* = 216) met the inclusion criteria (i.e., being ≥65 years, inhabitant of one of the selected wards, able to walk, independent in daily life, and mentally healthy) and received GPS tracking units. However, 43 participants were excluded because they had not used the tracking units. Thus, the final sample included 173 participants; 93 participants from low-deprivation areas and 80 participants from high-deprivation areas [13]. An assistant/translator assisted 58 participants who were not English speakers or who needed help to complete the questionnaire on individual characteristics [13].

### 2.3. Outdoor Walking Levels

Data on the location (x, y), date, and time of participants’ outdoor walking levels were collected using a GPS tracking unit (i-gotU GT-600 GPS data-logger, Mobile Action Technology Inc., New Taipei City, Taiwan) [13]. All participants were trained and used the units (set on motion detector mode and 2 s recording interval). The tracking unit was used for three to eight days (mean = 4.95, SD = 1.61). This period depended on participants’ willingness and availability for using the tracking unit. Collected data were imported to a GIS and each participant’s outdoor walking level was measured within a defined neighbourhood (a 2 km crow-fly buffer around each participant’s home). All outdoor walking activities within the defined neighbourhood were taken into account and each participant’s (average) outdoor walking level (minutes per day) was calculated as (sum of durations of all walking activities)/(number of days that participant was loaned the GPS device) [13].

### 2.4. Individual Characteristics

A questionnaire was used to collect data on the following individual characteristics: age (65–74 years old or 75 years old and over); gender; marital status (single or in relationship); ethnicity (black and minority ethnic (BME) groups—i.e., Asian, Black, or mixed ethnic heritage—or white British [55]); educational attainment (sub GCSE (General Certificates of Secondary Education or its equivalents) or GCSE and higher; and perceived health status over the last twelve months (poor or good)). Missing data on each variable—except education attainment with 11% missing data—were less than 5% [13].

### 2.5. Neighbourhood Green Spaces Characteristics

We used a GIS (ArcGIS 10.4, ESRI, Redlands, CA, USA) and data presented in Table 2 to objectively measure neighbourhood green spaces characteristics (i.e., proximity, attractiveness, size, and number).

**Figure 2:**
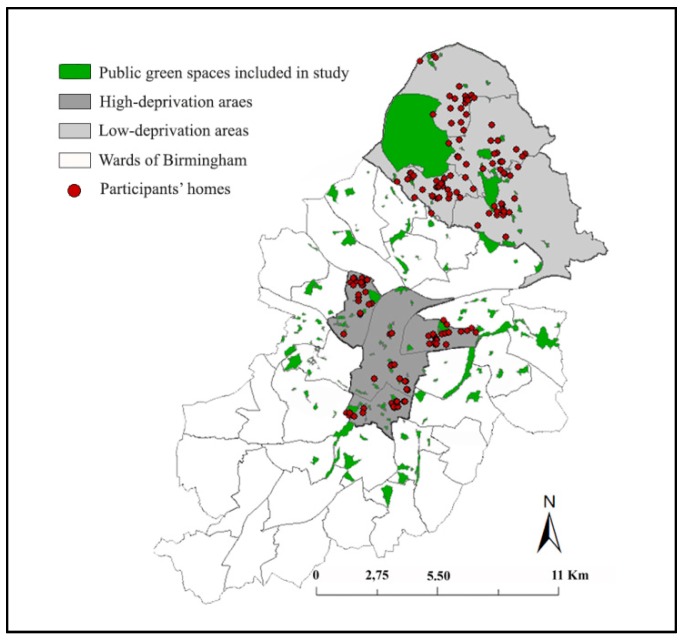
Participants’ homes in low- and high-deprivation areas (adapted from the work of [13]) and digitised public green spaces included in study (data from Birmingham City Council [56] and Ordnance Survey (OS) MasterMap data ©Crown Copyright/database right 2016. An Ordnance Survey/EDINA Digimap (the national data centre for U.K. academics) supplied service).

To identify neighbourhood green spaces, we used data on ‘open spaces’ provided by the Birmingham City Council [56]. Public parks and gardens, natural green spaces, and amenity green spaces were all taken into account [56]. Then, we used the ‘land theme’ of topography layer of Ordnance Survey MasterMap (OSMM) as a base-map and we digitised the boundaries of Birmingham green spaces. Green spaces’ gates were defined as points where the green space boundaries intersect with the ‘pedestrian route network’ (Table 2) [17]. Boundaries of all green spaces and location of green spaces gates were cross-referenced with Google Earth images. Similar to previous studies on walking [24,30], all Birmingham green spaces ≥ 2 acres (0.81 hectare) were included in the study, as smaller green spaces are not suitable for physical activity [57]. In total, 150 green spaces ≥2 acres were identified. For each participant, green spaces with at least one gate within the participant’s home-based neighbourhood were considered as neighbourhood green spaces (Figure 2).

To measure proximity, pedestrian route network distance (meter) was measured—as it better represents the true relevant spatial distance [17] than Euclidean distance [63,64]—and the distance between each participant’s home and the closest gate of each green space was calculated.

For measuring attractiveness, we used ‘park and conservation’ data (Table 2), and similar to a previous study [65], we considered the presence or absence of specific features in neighbourhood green spaces. Six features of attractiveness, which are important for older adults, were taken into account: café [32], lake or reservoir [32], toilet [32], wildlife [66], woodland [67], and farm or conservation park [66,68]. Data on the presence of lakes were cross-referenced with Google Earth images. Data on other green space features (e.g., lighting and safety, presence of bench, pavement, and so on) were not available. The presence and absence of each feature of attractiveness were scored as 1 and 0, respectively. We gave a minimum attractiveness score of 1 to all green spaces, as there is some greenery in all these green spaces. An overall score of attractiveness is usually used to study its association with walking [17]. We calculated the overall attractiveness as the sum of all attractiveness features scores (max. score = 6) and (minimum attractiveness) score of 1 allocated to each neighbourhood green space. We tested the internal consistency of the items (café, lake or reservoir, toilet, wildlife, woodland, and farm or conservation park). The reliability testing showed acceptable internal consistency, Cronbach’s *α* = 0.70, of this overall score. Therefore, the overall attractiveness score of each neighbourhood green space was between 1 and 7.

For size and number, we measured size as area (hectare) of land covered by each neighbourhood green space. We also counted number of neighbourhood green spaces available to each participant.

**Figure 3:**
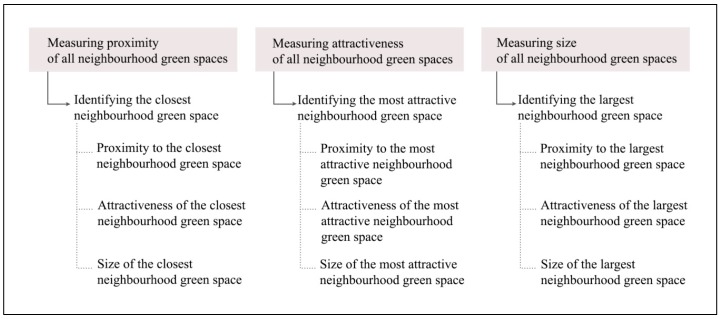
Identifying three types of neighbourhood green spaces and their characteristics.

Using measures of proximity, attractiveness, and size of all neighbourhood green spaces, we identified three types of neighbourhood green spaces (i.e., the closest, most attractive, and largest) for each participant [30] (Figure 3). Depending on the number of green spaces available to each participant within a 2 km radius (home-based neighbourhood) and their characteristics, the same green space could be simultaneously the closest, most attractive, and largest green space [30]. Measures of each type of neighbourhood green space characteristic (i.e., proximity, attractiveness, and size)—as well as the number of neighbourhood green spaces—were exported to statistical software (SPSS 24, IBM, Armonk, NY, USA) for statistical analyses.

### 2.6. Data Analysis

Descriptive statistics was applied to analyse the participants’ individual characteristics and outdoor walking levels. We used independent sample *t*-tests to compare outdoor walking levels between low- and high-deprivation areas (averaged GPS lending period (number of days) was not significantly different between low- and high-deprivation areas [13]).

Characteristics (i.e., proximity, attractiveness, and size) of each type of neighbourhood green space were compared between low- and high-deprivation areas using independent sample *t*-tests. For comparing the total number of neighbourhood green spaces between low- and high-deprivation areas, because we had count (over-dispersed) data, we generated negative binomial models and used the number of neighbourhood green spaces as the dependent variable and area deprivation as the factor.

In this study, we used nested data (participants from low- and high-deprivation areas). Therefore, we employed hierarchical (also known as multilevel) linear regression models to study relationships between characteristics (e.g., proximity, attractiveness, and size) of each type of neighbourhood green space (i.e., the closest, most attractive, and largest)—and also between the total number of neighbourhood green spaces—and outdoor walking levels. Correlations between the characteristics of three types of neighbourhood green spaces (i.e., the closest, most attractive, and largest) were tested and reported in Supplementary Materials Table S1. For each type of neighbourhood green space (i.e., the closest, most attractive, and largest), we examined each green space characteristic (e.g., proximity, attractiveness, and size) individually. Each model was tested for the interaction between the neighbourhood green space characteristic and area deprivation. When the interaction was significantly related to outdoor walking levels, analyses were done for low- and high-deprivation areas separately. Each model was also controlled for two individual characteristics (i.e., marital status and ethnicity), because participants’ outdoor walking was significantly related only to these two individual characteristics; participants who were in a relationship or white British walked more than their peers who were single or from the BME group [13]. No relationship was found between other individual characteristics (i.e., age, gender, educational attainment, health status) and participants’ outdoor walking levels [13]. Correlations between these two personal characteristics (i.e., marital status and ethnicity) and characteristics (e.g., proximity, attractiveness, and size) of three types of neighborhood green spaces (i.e., the closest, most attractive, and largest) were tested and reported in Supplementary Materials (Table S2). In all models, logarithmic transformation was applied on all variables (x + 1) to reduce heteroscedasticity. Moreover, the missing data were excluded listwise. A *p*-Value < 0.05 was considered as significant in all statistical analyses.

## 3. Results

### 3.1. Sample Characteristics and Participants’ Outdoor Walking Levels

The sample characteristics are presented in Table 3. Compared with participants in low-deprivation areas, a higher percent of participants from high-deprivation areas was from BME groups and/or had low educational attainment (sub-GCSE). Similar percentage of participants (over 90%) perceived a good health status in low- and high-deprivation areas.

Participants’ outdoor walking levels were between 0.00 and 68.33 min/day, with an average of 14.99 min/day. Participants living in high-deprivation areas spend less time on outdoor walks than those living in how-deprivation areas (Table 4).

### 3.2. Inequalities in Neighbourhood Green Spaces Characteristics

A summary of three types of neighbourhood green spaces (i.e., the closest, most attractive, and largest) in low- and high-deprivation areas is presented in Table 5. For the total sample of participants, 38, 9, and 10 green spaces were identified as the closest, most attractive, and largest neighbourhood green spaces, respectively. Greater numbers of the total, most attractive, and largest neighbourhood green spaces were identified for the sample from high-deprivation areas compared with those from low-deprivation areas.

Table 6 shows spatial inequalities in the characteristics of three types of neighbourhood green spaces (i.e., the closest, most attractive, and largest) between low- and high-deprivation areas. Participants living in high-deprivation areas have longer distances to the most attractive and the largest neighbourhood green spaces than their peers living in low-deprivation areas. Moreover, they have smaller and less attractive (all types of) neighbourhood green spaces than participants from low-deprivation areas. Nevertheless, participants from high-deprivation areas have shorter distances between their homes and the closest neighbourhood green spaces—and a higher number of green spaces (unstandardized coefficient = −0.67, standard error = 0.16, *p* = 0.000) in their neighbourhoods—than those from low-deprivation areas. All differences in neighbourhood green spaces characteristics between low- and high-deprivation areas are significant.

### 3.3. Relationships between Neighbourhood Green Spaces Characteristics and Outdoor Walking Levels

Table 7 shows the relationships between the characteristics of three types of neighbourhood green spaces (i.e., the closest, most attractive, largest) and participants’ outdoor walking levels. Only one neighbourhood green space characteristic (i.e., size) is significantly related to outdoor walking levels (Table 7). The size of three types of neighbourhood green spaces is positively related to participants’ outdoor walking levels. This means that participants who have bigger (the closest, most attractive, or largest) neighbourhood green space are more likely to take outdoor walks. There are no significant relationships between other characteristics of neighbourhood green spaces (i.e., proximity, attractiveness, and number of green spaces) and outdoor walking levels (Table 8). The relationships between neighbourhood green spaces characteristics and outdoor walking levels are similar in low- and high-deprivation areas (Table 7).

### 3.4. Combination of Results

Table 8 presents a combination of the results on spatial inequalities in neighbourhood green spaces characteristics between low- and high-deprivation areas and the results on relationships between neighbourhood green spaces characteristics and participants’ outdoor walking levels. It shows that ‘size’ is the only neighbourhood green space characteristic related to participants’ outdoor walking levels. Moreover, participants living in high-deprivation areas have smaller size of (the closest, most attractive, and largest) neighbourhood green spaces than those living in low-deprivation areas. Accordingly, spatial inequalities in the size of neighbourhood green spaces in high- versus low-deprivation areas may influence disparities in participants’ outdoor walking levels. Although there are spatial inequalities in other characteristics (i.e., attractiveness, proximity, and number) of neighbourhood green spaces in high- versus low-deprivation areas, these neighbourhood green spaces characteristics are not related to participants’ outdoor walking levels (Table 8).

## 4. Discussion

This study examined inequalities in neighbourhood green spaces characteristics (i.e., proximity, attractiveness, size, and number) in high- versus low-deprivation areas and their possible influences on older adults’ outdoor walking levels in Birmingham, United Kingdom. Similar to previous studies [8,9,13], it showed that participants living in high-deprivation areas walk less than those living in low-deprivation areas. It also showed that inequalities in the size of neighbourhood green spaces in high- versus low-deprivation areas might influence disparities in participants’ outdoor walking levels. These findings are discussed in the following subsections.

### 4.1. Neighbourhood Green Spaces’ Size

This study demonstrated that the size of neighbourhood green spaces is related to participants’ outdoor walking levels. Therefore, participants who have larger (but not necessarily close and attractive) neighbourhood green spaces are more likely to walk outside their homes. Similar to previous studies on walking [24,30], this study showed that mere proximity or attractiveness of neighbourhood green spaces does not encourage participants to take outdoor walks. Nearby or attractive neighbourhood green spaces that are larger are conductive to undertaking outdoor walking. Therefore, this study supports previous studies highlighting the importance of the size of green spaces for walking and use of green spaces [19,24,30,34,69,70,71] and shows that the relationship between the size of neighbourhood green spaces and outdoor walking levels holds true for older adults.

The findings also show that neighbourhood green spaces are smaller in high-deprivation areas than in low-deprivation areas. Participants living in high-deprivation areas also need to take a longer distance to get to the largest neighbourhood green space than those living in low-deprivation areas. These findings support previous studies on spatial inequalities in the size of green spaces [27,72] in high- versus low-deprivation areas. Given the relationship between the size of neighbourhood green spaces and outdoor walking levels, these findings provide a possible explanation for lower outdoor walking levels in high- versus low-deprivation areas.

Accordingly, to encourage outdoor walking among participants living in high-deprivation areas, policy makers, particularly those involved in urban planning and design, may need to enhance the distribution of larger neighbourhood green spaces in high-deprivation areas. However, creating large green spaces in high-deprivation areas located in the inner part of the city—where the city is often compact and the land is scarce and expensive—is the most complicated and expensive strategy [31]. Haaland et al. [73] have discussed this issue and suggested possible solutions for this challenge. One possible solution could be creating green networks: linking existing (and new) neighbourhood green spaces through green streets/corridors [73]. To this end, it may be necessary to change the way land is valued [74]. For example, brownfield land of the inner city could be valued in relation to its health services potential to create a green network, rather than in relation to its potential for real estate or regeneration profits [75]. This research did not include green networks, owing to unavailability of data on green corridors. Future studies may consider this issue to study the influence of inequalities in neighbourhood green spaces on outdoor walking among different population subgroups, including older adults.

Nevertheless, it is worthy to mention that this study focused on the population of older adults. Smaller neighbourhood green spaces may have other benefits for certain sectors of the population (e.g., parents with small kids) that are beyond the scope of this study [34]. Future studies may explore how different sizes of neighbourhood green spaces influence different subgroups’ activities.

### 4.2. Neighbourhood Green Spaces’ Proximity, Attractiveness, and Number

Neighbourhood green spaces’ proximity is not related to outdoor walking levels in this study. This result is consistent with some previous studies on adults’ physical activity [19,76] or use of green space [77]. Some older adults may use other modes of travel (e.g., car or public transport) to reach nearby neighbourhood green spaces, in order to save their energy for taking recreational walks within neighbourhood green spaces [27]. Moreover, although proximity is generally related to walking [18], for older adults’ outdoor walking, aesthetics, safety (particularly for women [13]), and condition of walking paths are also important [13,78]. They may not walk short distances because of the poor quality of a route (e.g., a noisy and unsafe route). Moreover, objective and subjective measures of proximity have different impacts on physical activity [79]. This study used objective measure of proximity. Disparate results might be observed if subjective measures of proximity to neighbourhood green spaces are used [76]. This study faced a lack of data on the quality of routes that are traversed by participants and on subjective measures of proximity to neighbourhood green spaces. Future research may use both objective and subjective measures of proximity to neighbourhood green spaces, to address the quality of routes and to get more comprehensive results.

In this study, consistent with previous research [71,76], the attractiveness of neighbourhood green spaces is not related to outdoor walking levels. It can be explained by the limited number of features involved in measuring the overall attractiveness of neighbourhood green spaces in this study. Evidence indicates that some features of attractiveness—such as lighting, signs of vandalism, noise, maintenance, and cleanness [26,32,66]—are important for older adults’ use of neighbourhood green spaces. Moreover, different features of attractiveness may not be equally important for older adults and more sophisticated methods of weighing features may be required before deriving an overall score of neighbourhood green spaces’ attractiveness [24]. Giles-Corti et al. [24] have applied such methods to measure the overall attractiveness of green spaces in their study of adults’ walking. The present study, however, did not employ this method because of a lack of data on more features of attractiveness (e.g., lighting, safety, noise) and on the importance of each feature for participants. This is worthy for future research on older adults’ outdoor walking to address this issue.

There is no relationship between the number of neighbourhood green spaces and outdoor walking levels in this study. Therefore, the mere existence of neighbourhood green spaces is not sufficient to encourage older adults to walk. Other characteristics of neighbourhood green spaces (especially size) need to be taken into account in developing strategies on providing neighbourhood green spaces. Moreover, Jane Jacobs [80] discussed that, although neighbourhood green spaces have benefits, they could be also harmful to safety and well-being if they are not well-designed. In her view, people do not use neighbourhood green spaces just because they are there. The location of neighbourhood green spaces (e.g., in areas with multiple uses) and attractive activities (e.g., events and social /cultural activities) determine the use of neighbourhood green spaces [80]. Future research may wish to address these aspects of neighbourhood green spaces.

This study also demonstrated spatial inequalities in proximity, attractiveness, and number of neighbourhood green spaces in high-versus low-deprivation areas. Consistent with some previous studies [81,82], it showed that there are more neighbourhood green spaces in high- versus low-deprivation areas. However, these neighbourhood green spaces are less attractive and smaller than those in low-deprivation areas. These findings are generally consistent with previous studies that show fewer aesthetic features or natural elements [31,42] and fewer amenities [42,82,83] in neighbourhood green spaces in high-deprivation areas. Moreover, this study showed that participants living in high-deprivation areas need to go a longer distance to get to the most attractive or largest neighbourhood green spaces than those living in low-deprivation areas. However, these spatial inequalities in high- versus low-deprivation areas do not drive disparities in participants’ outdoor walking levels, because relationships between neighbourhood green spaces proximity, attractiveness, and number and outdoor walking levels are insignificant.

### 4.3. Limitations

This study has several limitations: it is a cross-sectional study and is unable to draw causal relationships between neighbourhood green spaces characteristics and outdoor walking levels. As mentioned earlier, a limited number of unweighted attractiveness features was included in this study owing to a lack of data on more features of attractiveness and their importance for participants. As explained before, only objectively measured proximity was included in this study, owing to the unavailability of a subjective measure of this neighbourhood green space characteristic. The study may be subject to self-selection bias; that is, people interested in walking may choose to reside in neighbourhoods that support walking. This study was done in one U.K. city and used a convenience sampling approach; thus, participants may not be representative of all older citizens (e.g., older citizens with poor health status). Moreover, a small sample size was used in this study; thus, findings of this study may not be generalizable. Nevertheless, the findings of this study are consistent with some previous research. Moreover, this study is among the first studies investigating the influence of spatial inequalities in neighbourhood green spaces characteristics in high- versus low-deprivation areas on disparities in older adults’ (objectively measured) outdoor walking levels. Therefore, it provides a new insight into the spatial inequalities and older adults’ physical activity, which is applicable to more heterogeneous samples, as well as other cities and research.

## 5. Conclusions

This study contributes to the limited, though growing and important, body of literature investigating the influence of neighbourhood green spaces characteristics on older adults’ physical activity. Unique aspects of this study are focusing on older adults, a relevant, but less-studied population; addressing spatial inequalities in neighbourhood green spaces characteristics between low- and high-deprivation areas and their influences on outdoor walking; and using objectively (GPS) measured outdoor walking levels. It showed that the size of neighbourhood green spaces is related to participants’ outdoor walking. It also showed that there are inequalities in the size of neighbourhood green spaces in high- versus low-deprivation areas. Accordingly, inequalities in the size of neighbourhood green spaces in high- versus low-deprivation areas may influence the disparities in participants’ outdoor walking levels. This study may help to develop policies and guidelines on creating suitable neighbourhood green spaces that support older adults’ outdoor walking and assist design and management interventions related to neighbourhood green spaces characteristics. Strategies for creating larger neighbourhood green spaces in high-deprivation areas may encourage outdoor walking among older residents of these areas.

## Figures and Tables

**Table 1:** Four neighbourhood green spaces characteristics.

Characteristic	Definition
Proximity	The closest geographic distance between people’s homes and a neighbourhood green space, regardless of the green space’s size or attractiveness [31].
Attractiveness	The internal characteristic of the green space that includes various features related to environmental quality (e.g., presence of a water feature and biodiversity), amenity (e.g., presence of toilet [32] and presence of walking paths), and safety (e.g., lighting and quietness) [17,24]. This characteristic is also known as quality of green space [31].
Size	The area (e.g., hectare) of a neighbourhood green space [31].
Number	The total number of available green spaces within a neighbourhood [31].

**Table 2:** Data used for objective measures of neighbourhood green spaces characteristics.

ND Green Spaces Characteristic	Data	Data Definition	Source
Proximity	Pedestrian route network ^a^	A U.K. route network that includes drivable roads and urban paths suitable for non-vehicular users (i.e., all man-made footpaths, subways, steps, foot bridges, and cycle paths) [14]. It does not include forbidden routes for pedestrians (motorways and slip roads) in the United Kingdom [58].	A previous study on the United Kingdom [14]
Attractiveness	Parks and nature conservation	A list of green spaces of Birmingham and information on amenities and facilities provided in these green spaces.	Birmingham City Council [59,60,61]
Size and number	Open spaces ^a^	Map of private and public green spaces of each ward of Birmingham (in pdf format).	Birmingham City Council [56]
	Topography layer of OSMM 2016a: The land theme ^a^	The man-made and natural features that delineate and describe the surface cover (except routes of communication and buildings). It includes parks; playing fields; football pitches; golf courses; slopes and cliffs; car parks; gardens; woodlands; and other areas of vegetation, including scrub, heath, rough grass, and marshland [62].	Digimap/EDINA

Note. ^a^ These data were also used for identifying neighbourhood green spaces. ND = neighbourhood; Digimap/EDINA = the national data centre for U.K. academics; OSMM = Ordnance Survey MasterMap.

**Table 3:** Sample characteristics in low- and high-deprivation areas and in total.

Participants’ Characteristics	Low-Deprivation Areas	High-Deprivation Areas	Total Sample
Number of participants	93	80	173
Average age of participants (M (SD))	74.8 (5.82)	73.5 (5.95)	74.2 (5.90)
Age (%)			
75 years old and over	53	43	48
65–74 years old	47	57	52
Gender (%)			
Men	30	59	43
Women	70	41	57
Marital status (%):			
In relationship	53	53	53
Single	47	47	47
Ethnicity (%):			
White British	97	41	71
BME groups	3	59	29
Educational attainment (%):			
GCSE and higher	80	24	54
Sub-GCSE	10	64	35
Health status (%):			
Good	93	92	92
Poor	6	8	7

Note. Data from the work of [13]. GCSE, General Certificates of Secondary Education.

**Table 4:** Disparities in outdoor walking levels between low- and high-deprivation areas.

	M	SD	t	Cohen’s *d*	Effect Size *r*
Low-deprivation areas	17.05	14.51	*t*(171) = 1.98 *	0.30	0.15
High-deprivation areas	12.60	14.97			

Note. *M* = mean; *SD* = standard deviation; *t* = t-test value. Data from the work of [13]. * *p* < 0.05.

**Table 5:** Type of neighbourhood green spaces available to participants in low- and high-deprivation areas and in total.

Type of Neighbourhood Green Space	Low-dep.	High-dep.	Total
The closest (*N*) ^a^	21	17	38
The most attractive (*N*) ^a^	2	7	9
The largest (*N*) ^a^	2	8	10
Number of neighbourhood green spaces (*N*)	38	85	123

Note. ^a^ Some neighbourhood green spaces are simultaneously the closest, most attractive, and largest green space in neighbourhoods. Low-dep. = neighbourhood green spaces identified for the sample from low-deprivation areas; high-dep. = neighbourhood green spaces identified for the sample from high-deprivation areas; total = neighbourhood green spaces identified for the sample from low- and high-deprivation areas; *N* = number.

**Table 6:** Results of independent sample *t*-test: spatial inequalities in neighbourhood green spaces characteristics between low- and high-deprivation areas.

Type of Neighborhood Green Space/Characteristic	*M*		*SD*		*t*	Cohen’s *d*	Effect Size *r*
	Low.	High.	Low.	High.			
Closest/							
Proximity (m)	602.77	375.84	345.04	263.99	t (171) = 4.80 ***	0.74	0.35
Attractiveness	2.85	0.91	2.48	1.18	*t* (136) = 6.71 ***	1.00	0.45
Size (ha)	307.43	12.86	406.31	9.49	*t* (92) = 6.99 ***	1.03	0.46
M. attractive/							
Proximity (m)	1117.38	1366.47	673.22	851.15	t (150) = −2.11 *	−0.33	−0.16
Attractiveness	5.16	1.95	1.35	0.84	*t* (156) = 19.01 ***	2.86	0.82
Size (ha)	658.46	21.21	353.40	10.57	*t* (92) = 17.38 ***	2.55	0.77
Largest/							
Proximity (m)	1117.38	1704.66	673.23	747.32	t (171) = −5.44 ***	−0.83	−0.38
Attractiveness	5.16	1.14	1.35	1.05	*t* (169) = 21.97 ***	3.32	0.86
Size (ha)	658.46	31.54	353.40	9.42	*t* (92) = 17.10 ***	2.51	0.78

Note. *M* = mean; *SD* = standard deviation; *t* = t-test value; M. attractive = most attractive; Low. = low-deprivation areas; High. = high-deprivation areas. * *p* < 0.05; *** *p* < 0.001.

**Table 7:** Results of hierarchical regression: relationships between characteristics of three types of neighbourhood green spaces (the closest, most attractive, largest) and outdoor walking levels.

Type of Neighborhood Green Space/Characteristic	Outdoor Walking ^a^	Interaction	Low-dep. Areas ^b^	High-dep. Areas ^c^
	B (SE)	B (SE)	B (SE)	B (SE)
Closest/				
Proximity (m)	0.06 (0.10)	−0.70 (0.04)	-	-
Attractiveness	0.17 (0.12)	−0.36 (0.22)	-	-
Size (ha)	0.09 (0.04) *	−0.09 (0.08)	-	-
M. attractive/				
Proximity (m)	−0.11 (0.11)	−0.06 (0.03)	-	-
Attractiveness	0.29 (0.22)	−0.44 (0.21) *	0.72 (0.43)	−0.71 (0.47)
Size (ha)	0.11 (0.05) *	−0.09 (0.08)	-	-
Largest/				
Proximity (m)	−0.13 (0.12)	−0.05 (0.03)	-	-
Attractiveness	0.15 (0.14)	−0.50 (0.21) *	0.72 (0.43)	−0.40 (0.27)
Size (ha)	0.17 (0.07) **	0.04 (0.10)	-	-
Number of neighbourhood green spaces	−0.13 (0.21)	−0.22 (0.11) *	0.12 (0.32)	0.42 (0.55)

Note. ^a^ Outdoor walking levels; ^b^ outdoor walking levels of participants living in low-deprivation areas; ^c^ outdoor walking levels of participants living in high-deprivation areas. M. attractive = most attractive; interaction = relationship of interaction (between the neighbourhood green spaces characteristic and area deprivation) and outdoor walking levels; B = unstandardized coefficient, SE = standard error. * *p* < 0.05; ** *p* ≤ 0.01.

**Table 8:** Combination of results on spatial inequalities in neighbourhood green spaces characteristics and relationships between neighbourhood green spaces characteristics and outdoor walking levels.

Type of Neighborhood Green Space/Characteristic	Spatial Inequalities in Neighbourhood Green Space Characteristics	Relationship with Outdoor Walking Levels
Closest/		
Proximity (m)	Low-dep. > High-dep.	No
Attractiveness	Low-dep. > High-dep.	No
Size (ha)	Low-dep. > High-dep.	Yes
M. attractive/		
Proximity (m)	Low-dep. < High-dep.	No
Attractiveness	Low-dep. > High-dep.	No
Size (ha)	Low-dep. > High-dep.	Yes
Largest/		
Proximity (m)	Low-dep. < High-dep.	No
Attractiveness	Low-dep. > High-dep.	No
Size (ha)	Low-dep. > High-dep.	Yes
Number of neighbourhood green spaces	Low-dep. < High-dep.	No

Note. Low-dep. = low-deprivation areas; High-dep. = high-deprivation areas; Yes = the neighbourhood green space characteristic is related to outdoor walking levels; No = the neighbourhood green space characteristic is not related to outdoor walking levels.

## Supplementary Materials

The following are available online at https://www.mdpi.com/1660-4601/16/22/4379/s1, Table S1: Correlations between characteristics of three types of neighbourhood green spaces (i.e., the closest, most attractive, and largest); Table S2: Correlations between participants’ personal characteristics and characteristics of three types of neighbourhood green spaces (i.e., the closest, most attractive, and largest).
