# Supplementary material for: Older Adults’ Outdoor Walking and Inequalities in Neighbourhood Green Spaces Characteristics
Source: Int J Environ Res Public Health. 2019 Nov 9;16(22):4379. doi: 10.3390/ijerph16224379 (PMC6888485; doi:10.3390/ijerph16224379)
Supplement: Supplementary file 1 [file ijerph-16-04379-s001.pdf]

International Journal of
Environmental Research and Public Health

MDPI

Article

Older Adults' Outdoor Walking and Inequalities in Neighbourhood Green Spaces Characteristics

Razieh Zandieh, Javier Martinez, and Johannes Flacke

Table S1: Correlations between characteristics of three types of neighbourhood green spaces (i.e., the closest, most attractive, and largest).

Type of green space / characteristic	Closest / attractiveness	Closest / size	Most attractive / proximity	Most attractive / attractiveness	Most attractive / size	Largest / proximity	Largest / attractiveness	Largest / size
Closest / Proximity	0.32<br>*0.000*	0.23<br>0.003	0.06<br>*0.438*	0.36<br>*0.000*	0.37<br>*0.000*	0.04<br>*0.572*	0.32<br>*0.000*	0.37<br>*0.000*
Attractiveness		0.88<br>*0.000*	- 0.35<br>*0.000*	0.52<br>*0.000*	0.48<br>*0.000*	- 0.45<br>*0.000*	0.47<br>*0.000*	0.48<br>*0.000*
Size			- 0.37<br>*0.000*	0.56<br>*0.000*	0.59<br>*0.000*	- 0.46<br>*0.000*	0.54<br>*0.000*	0.59<br>*0.000*
Most attractive / Proximity				0.07<br>*0.331*	- 0.04<br>*0.563*	0.65<br>*0.000*	0.08<br>*0.931*	- 0.05<br>*0.549*
Attractiveness					*0.95*<br>*0.000*	- 0.23<br>*0.003*	0.97<br>*0.000*	0.95<br>*0.000*
Size						- 0.22<br>*0.004*	0.93<br>*0.000*	1.00<br>*0.000*
Largest / Proximity							- 0.29<br>*0.000*	- 0.21<br>*0.005*
Attractiveness								0.93<br>*0.000*

Note. This table shows Pearson correlation values; p-values are in *italic*.

Table S2: Correlations between participants' individual characteristics and characteristics of three types of neighbourhood green spaces (i.e., the closest, most attractive, and largest).

Participants' individual characteristics	Type of green space / characteristic								
	Closest / proximity	Closest / attractiveness	Closest / size	Most attractive / proximity	Most attractive / attractiveness	Most attractive / size	Largest / proximity	Largest / attractiveness	Largest / size
Marital status	- 0.16<br>*0.039*	- 0.08<br>*0.301*	0.05<br>*0.532*	0.05<br>*0.494*	0.02<br>*0.769*	0.01<br>*0.908*	0.01<br>*0.943*	0.02<br>*0.812*	0.01<br>*0.890*
Ethnicity	- 0.15<br>*0.043*	- 0.22<br>*0.003*	- 0.26<br>*0.000*	0.18<br>*0.019*	- 0.49<br>*0.000*	- 0.48<br>*0.000*	0.15<br>*0.048*	- 0.52<br>*0.000*	- 0.47<br>*0.000*

Note. This table shows Pearson correlation values; p-values are in *italic*.
